# Magnetic resonance fistulography with percutaneous jelly: A novel and cost-effective technique

**DOI:** 10.4102/sajr.v29i1.3166

**Published:** 2025-08-15

**Authors:** Umamaheshwari K. Basavaraju, Shivani S. Ravate Patil, N. Manupratap, B. Tejesh, Shubha Tavarakere Shamasundara, Larryth Reuben

**Affiliations:** 1 Department of Radiodiagnosis, Mysore Medical College and Research Institute, Mysore, India

**Keywords:** fistula-in-ano, aqueous gel instillation, MR fistulogram, internal openings, secondary ramification

## Abstract

**Background:**

Magnetic resonance fistulography (MRF) is a key non-invasive imaging technique for mapping perianal fistulas before surgery. The instillation of aqueous jelly, like ultrasound gel, enhances the signal-to-noise ratio, improving image quality and MRF accuracy. This low-cost approach improves accessibility, especially in resource-limited settings, while ensuring patient comfort and safety.

**Objectives:**

To determine the impact of aqueous jelly on the accuracy of MRF in identifying fistulous tracts, internal openings, secondary ramifications and abscesses and the quality of visualisation of MRF with and without jelly instillation.

**Method:**

A cross-sectional study at Krishna Rajendra and Cheluvamba Hospitals, Mysore (October 2024–March 2025), included 40 patients with perianal fistulas. Magnetic resonance fistulography was performed using a 1.5 Tesla uMR 570 system, without and with 5 mL – 7 mL of sterile aqueous jelly instilled percutaneously. MRI findings were compared with intraoperative results. Data were analysed using Statistical Package for the Social Sciences (SPSS) to assess sensitivity, specificity and accuracy.

**Results:**

Magnetic resonance fistulography with gel improved visualisation, identifying internal openings in 92.5% of cases (vs. 24% without gel) and secondary tracts in 40% (vs. 17.5%). The accuracy compared to surgery was 97.5% for internal openings, 95% for secondary tracts, 97.5% for abscesses and 100% for supralevator extension.

**Conclusion:**

Aqueous jelly instillation enhances MRF accuracy, improving fistula visualisation and aiding in preoperative planning. This technique reduces recurrence and incontinence risk and supports more accurate surgical interventions.

**Contribution:**

This study re-emphasises the value of aqueous jelly in improving diagnostic accuracy and accessibility, especially in resource-limited settings.

## Introduction

Perianal fistula is a commonly encountered condition in routine surgical practice, with a significant impact on patient quality of life and healthcare systems. Accurate pre-surgical mapping of fistulous tracts is essential to prevent recurrence and guide effective surgical management. Magnetic resonance fistulography (MRF) is a non-invasive imaging technique that utilises MRI to visualise and evaluate complex anorectal or perianal fistulas. Magnetic resonance fistulography provides high-resolution images that accurately depict fistulous tract anatomy, facilitating precise surgical planning and improved patient outcomes.^1,2^

There is a paramount need for cost-effective, accurate and accessible diagnostic techniques, particularly in resource-limited settings. Recurrence of perianal fistulas because of inadequate preoperative assessment leads to repeated surgical interventions, prolonged patient discomfort and increased healthcare expenditures. Standard contrast agents used in imaging are expensive and may not always be available in low-resource settings. The introduction of aqueous jelly as a contrast medium presents a cost-effective alternative, reducing the overall financial burden of MRF while maintaining high diagnostic accuracy.^3,4^ Furthermore, improving diagnostic accuracy through enhanced visualisation can lead to better clinical decision-making and reduced recurrence rates, ultimately benefiting both patients and healthcare providers.^5,6^

While MRF is an established imaging modality, current contrast-enhanced techniques rely on traditional agents that may not be widely accessible or cost efficient. Percutaneous instillation of aqueous jelly, such as ultrasound gel, into the fistulous tract enhances tract visualisation by improving the signal-to-noise ratio, leading to clearer imaging.^7,8^ Despite its potential advantages, there is limited research on the efficacy of aqueous jelly in MRF imaging. This study aimed to bridge this knowledge gap by systematically evaluating the role of aqueous jelly as a contrast medium for MRF and assessing its impact on diagnostic accuracy, cost-effectiveness and patient experience.^9,10^

This study was based on the principles of MRI physics and contrast enhancement techniques. The effectiveness of aqueous jelly in MRF is based on its ability to fill fistulous tracts and provide a clear contrast to the surrounding soft tissues. The contrast enhancement principle follows the mechanism of signal amplification, owing to differences in tissue density and fluid composition. Previous studies on contrast media in MRI suggest that a well-distributed contrast agent can significantly enhance tract delineation.^11,12^ By integrating these imaging principles with practical clinical applications, this study aimed to establish a simplified, yet effective, approach to fistula imaging.

The study assessed the impact of aqueous jelly instillation on MRF accuracy in identifying fistulous tracts, internal openings, secondary ramification and abscesses, through comparing the visualisation quality of fistulous anatomy with and without aqueous jelly instillation.

## Research methods and design

A cross-sectional, retrospective study was conducted at Krishna Rajendra Hospital and Cheluvamba Hospital, Mysore, under the Department of Radiodiagnosis, Mysore Medical College and Research Institute (MMCRI). All patients with clinically suspected fistula-in-ano (new and postoperative cases), with a confirmed diagnosis via other imaging modalities such as ultrasonography (USG) or CT, and patients with active discharge suitable for aqueous jelly instillation were included. Patients with contraindications to MRI (e.g. pacemakers, metallic implants), known allergy to contrast agents or aqueous jelly components and severe perianal infections requiring urgent intervention before imaging, were excluded.

The study was hospital-based, conducted over 6 months from 01 October 2024 to 31 March 2025, using a purposive sampling technique. The sample size calculation was based on diagnostic performance metrics from Jat et al.’s^13^ study on MRI in perianal fistulas. Using a 95% confidence level and a 10% margin of error, sensitivity-based calculations required 14 participants, while specificity-based calculations required 39. To ensure statistical power and account for potential withdrawals, a final sample size of 40 participants was selected.

There were no intervention and comparison groups in this study. MRI fistulography was performed with and without aqueous jelly instillation, allowing for intra-patient comparisons of imaging quality. MRI of the perianal region was performed using a 1.5 Tesla uMR 570 system. A phased-array coil was used based on patient tolerance and anatomical requirements. Standard MRI sequences were acquired, including T1-weighted, T2-weighted and short tau inversion recovery (STIR) with and without aqueous jelly instillation. A strict aseptic technique was used for the MR fistulography technique. The area was cleaned with povidone-iodine, and sterile gloves and equipment were used. Anaesthesia was usually not required, as the procedure was minimally painful. A 22G plastic cannula was inserted into the external opening, and 5 mL – 7 mL of aqueous gel was slowly instilled to fill the tract, avoiding overdistension or leakage. The cannula was removed after instillation.

### Imaging parameters assessed

A hyperintense tract observed on T2-weighted and fat-saturated images in the anal and perianal regions, particularly in relation to the sphincter complex, was identified as the primary fistulous tract. Any additional hyperintensity extending beyond this tract was considered adjacent inflammation.^14,15^ The internal opening was determined using the anal clock, where the 12 o’clock position corresponded to the anterior aspect and the 6 o’clock position to the posterior aspect. Axial imaging was used to identify the precise location of these openings. A primary fistulous tract with secondary extensions, additional tracts or abscesses was classified based on anatomical location, including ischio-anal, intersphincteric or supralevator regions.^16^ To distinguish fistulous tracts from abscesses, the criteria outlined by Lunniss et al.^17^ were applied. According to these criteria, fistulas appear as fluid-filled tubular structures with a diameter of less than 10 mm, whereas abscesses have a diameter exceeding 10 mm. Any potential supralevator extension was also documented. The classification of the fistula type was based on the St. James’s University Hospital MRI classification system of Peri-Anal Fistula^18^:

- **Grade 1:** Simple linear intersphincteric fistula. This type of fistula follows a straightforward path from the perineal or natal skin cleft to the anal canal. The ischiorectal and ischioanal fossae remain unaffected.
- **Grade 2:** Intersphincteric fistulas with abscess or secondary tracts. These fistulas remain confined within the external sphincter but are accompanied by an abscess or secondary tract. Secondary tracts may take a horseshoe shape, crossing the midline, or they may branch within the ipsilateral intersphincteric plane.
- **Grade 3:** Transphincteric fistulas. This type penetrates both layers of the sphincter complex before curving downward to the skin via the ischiorectal and ischioanal fossae.
- **Grade 4:** Transphincteric fistulas with abscess or secondary tracts. A trans-sphincteric fistula can become complicated by infections within the ischiorectal or ischioanal fossa.
- **Grade 5:** Supralevator or translevator extension. In rare instances, perianal fistulous disease extends above the levator ani muscle insertion.

Patient data, including history and clinical findings, were recorded using a structured research proforma. Informed consent was obtained from all participants before the procedure. MRI findings, including internal openings, secondary ramification and abscesses, were compared with intraoperative findings. Data were recorded in Microsoft Excel and validated for consistency before analysis.

### Data analysis

Sensitivity, specificity and accuracy were computed using contingency tables, comparing MRI findings with intraoperative confirmation as the reference standard. Sensitivity was defined as the proportion of true positive cases correctly identified by MRI. Specificity was the proportion of true negatives correctly classified. Accuracy was the proportion of correctly classified cases among all cases studied. Data analysis was performed using SPSS (Statistical Package for the Social Sciences) version 24. A *p*-value < 0.05 was considered statistically significant.

### Ethical considerations

This cross-sectional study was approved by the Institutional Ethics Committee, MMCRI and Associated Hospitals, Mysore. Ethical committee clearance was obtained on 20 January 2025. Written informed consent was obtained from all patients prior to participation. Institutional review board approval number: MMC EC 106.25.

## Results

A total of 40 patients with fistula-in-ano were studied, both with and without aqueous gel instillation, ranging in age from 15 years to 60 years, with a mean age of 34.65 years. Among them, 28 were male patients and 12 were female patients.

**FIGURE 1:**
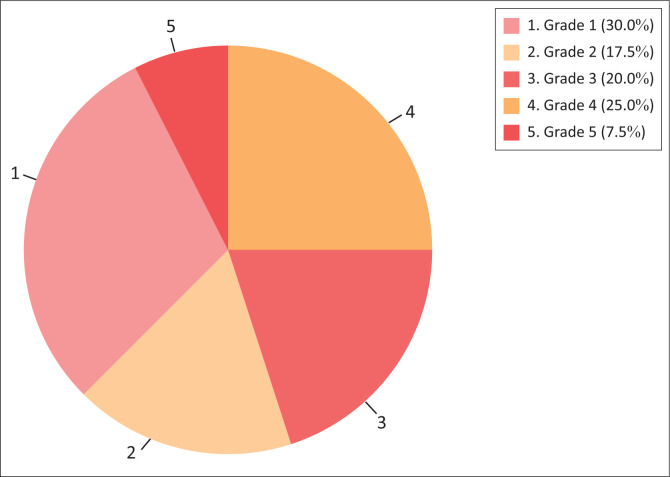
Pie chart demonstrating distribution of patients according to Grade of Fistulas in the study based on the St. James’s University Classification system.

Based on the St. James’s University Classification system,^18^ cases were categorised radiologically based on their complexity (Figure 1).

### Magnetic resonance fistulogram with and without aqueous gel instillation and surgical correlation for internal openings

**FIGURE 2:**
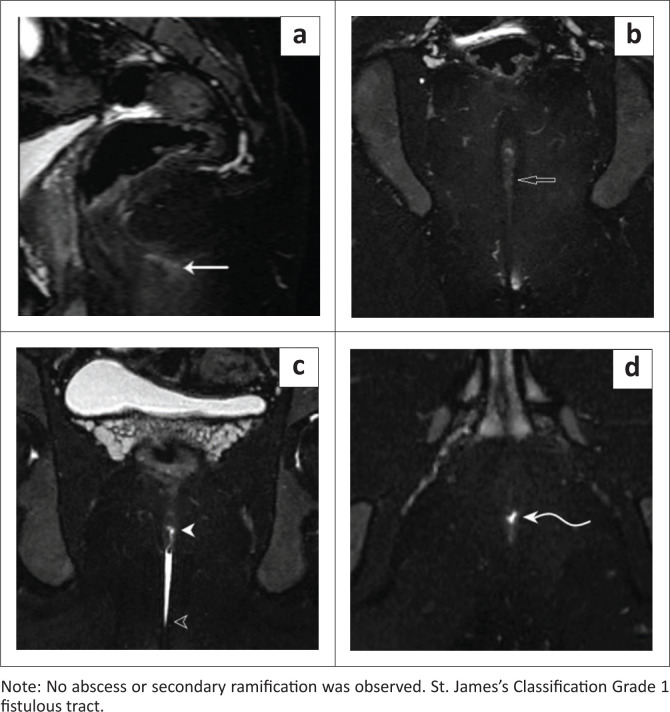
Sagittal T2 Fast Spin Echo (FSE)-weighted image pre-aqueous gel instillation demonstrating: (a) a T2 FSE hyperintense fistulous tract coursing through the intersphincteric plane (solid arrow); (b) Coronal T2 FSE-weighted image pre-aqueous gel instillation demonstrating the same (clear arrow); (c) Coronal T2 FSE-weighted image post-aqueous gel instillation demonstrating a well-delineated hyperintense fistulous tract in the intersphincteric plane with an internal opening (solid arrowhead) and external opening (clear arrowhead); (d) Axial T2 FSE-weighted image post-aqueous gel instillation demonstrating the internal opening in the intersphincteric plane (curved solid arrow).

**FIGURE 3:**
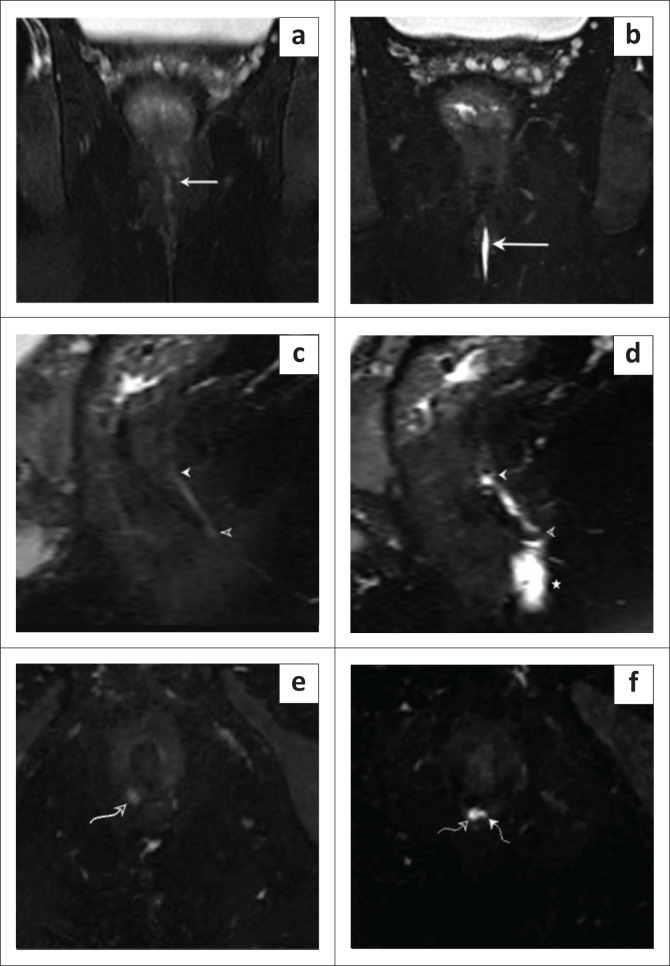
Coronal T2 FSE-weighted image: (a) pre and (b) post-aqueous gel instillation demonstrating a T2 hyperintense fistulous tract coursing through the intersphincteric plane (solid arrows). Sagittal T2 FSE-weighted image: (c) pre- and (d) post-aqueous gel instillation demonstrating a T2 hyperintense fistulous tract in the intersphincteric plane with an internal opening (solid arrowhead) and an external opening (hollow arrowhead). (d) A small abscess was also observed in the perianal region (asterisk) with post-aqueous gel instillation. Axial T2 FSE-weighted image (e) pre- and (f) post-aqueous gel instillation demonstrating the internal opening in the intersphincteric plane (curved hollow arrow) and a secondary ramification (curved solid arrow) – St. James’s Classification Grade 2 fistulous tract.

**FIGURE 4:**
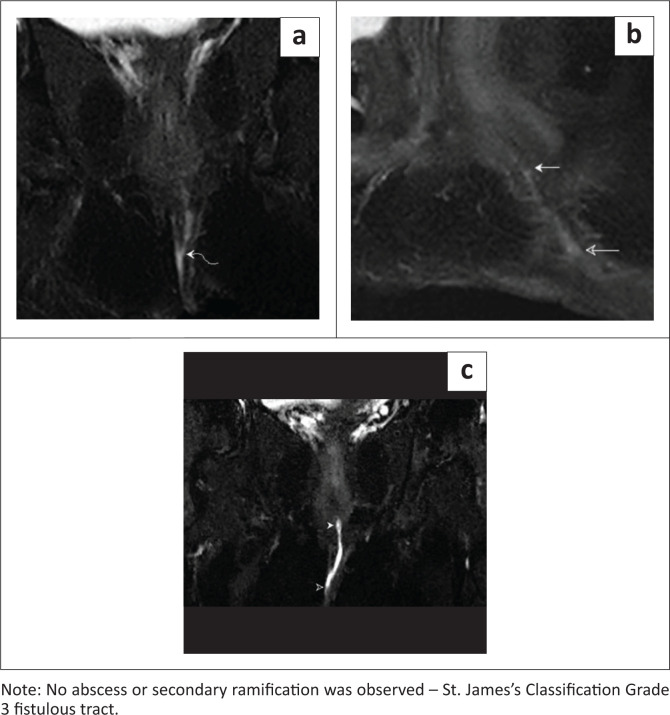
Coronal T2 FSE-weighted image pre-aqueous gel instillation demonstrating: (a) a fairly defined T2 hyperintense fistulous tract (curved solid arrow); (b) Sagittal T2 FSE-weighted image pre-aqueous gel instillation demonstrating the same (solid and hollow arrows); (c) Coronal T2 FSE-weighted image post-aqueous gel instillation demonstrating a well-delineated hyperintense tract piercing the external and internal anal sphincters with an internal opening at the 12 o’clock position – 1 o’clock position (solid arrowhead) and an external opening at the 12 o’clock position (hollow arrowhead).

**FIGURE 5:**
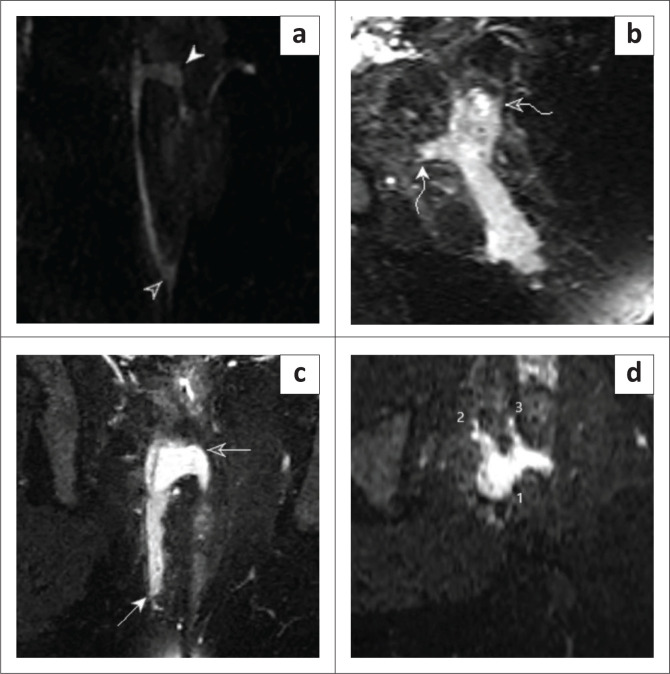
Coronal T2 FSE-weighted image pre-aqueous gel instillation demonstrating: (a) a hyperintense fistulous tract coursing through the ischioanal fossa with a small abscess (solid arrow head) and an external opening (hollow arrow head); (b) Sagittal T2 FSE-weighted image post-aqueous gel instillation demonstrating the abscess (curved hollow arrow) and internal opening (curved solid arrow); (c) Coronal T2 FSE-weighted image post-aqueous gel instillation demonstrating a well-delineated hyperintense tract in the ischioanal fossa piercing the external and internal anal sphincters with an internal opening at the 5–6 o’clock position, abscess (hollow arrow) and an external opening (solid arrow). (d) Axial STIR FSE image post-aqueous gel instillation demonstrating an abscess (1) with secondary ramifications (2,3) – St. James’s Classification Grade 4 fistulous tract.

Internal openings were visualised in 92.5 % of cases on MRF with aqueous gel instillation (Figure 2, Figure 3, Figure 4 and Figure 5). For the detection of internal openings, MRI demonstrated a sensitivity of 92.5%, a specificity of 100% and an accuracy of 97.5%, indicating that it was highly accurate. In contrast, without aqueous gel instillation, internal openings were successfully identified in only 24% of cases, highlighting the importance of gel instillation for optimal visualisation and diagnostic accuracy. The difference in detection rates between MRI with and without gel instillation was statistically significant (*p* < 0.001, Chi-square test).

### Magnetic resonance fistulogram with and without aqueous gel instillation and surgical correlation for secondary tracts

Magnetic resonance fistulogram with aqueous gel instillation significantly improved the detection of secondary tracts. Using this technique, secondary tracts were identified in 40% of cases, with MRI demonstrating a sensitivity of 88.89%, specificity of 91.67% and an accuracy of 95% in detecting these tracts. Although slightly lower than the detection of internal openings, MRI still demonstrated a high level of diagnostic performance. (Figure 3 and Figure 5) In contrast, without aqueous gel instillation, secondary tracts were identified in only 17.5% of patients. The *p*-value for the difference in detection rates of secondary tracts between MR fistulogram with and without aqueous gel instillation was statistically significant (*p* = 0.0481, Chi-square test).

### Magnetic resonance fistulogram with and without aqueous gel instillation and surgical correlation for abscess

Magnetic resonance fistulogram with aqueous gel instillation reliably improved the detection of abscesses. (Figure 3 and Figure 5). Using this technique, abscesses were identified in 50% of cases, with MRI demonstrating a sensitivity of 95.24%, specificity of 95% and an accuracy of 97%. In comparison, without aqueous gel instillation, abscesses were identified in only 47.5% of patients, highlighting the enhanced diagnostic precision provided by gel instillation for detecting abscesses. Although MRI with aqueous gel instillation showed a slightly higher detection rate for abscesses (50%) compared to without gel (47.5%), the difference was not statistically significant (*p* = 1.0, Chi-square test).

### Magnetic resonance fistulogram with and without aqueous gel instillation and surgical correlation for supralevator extension

**FIGURE 6:**
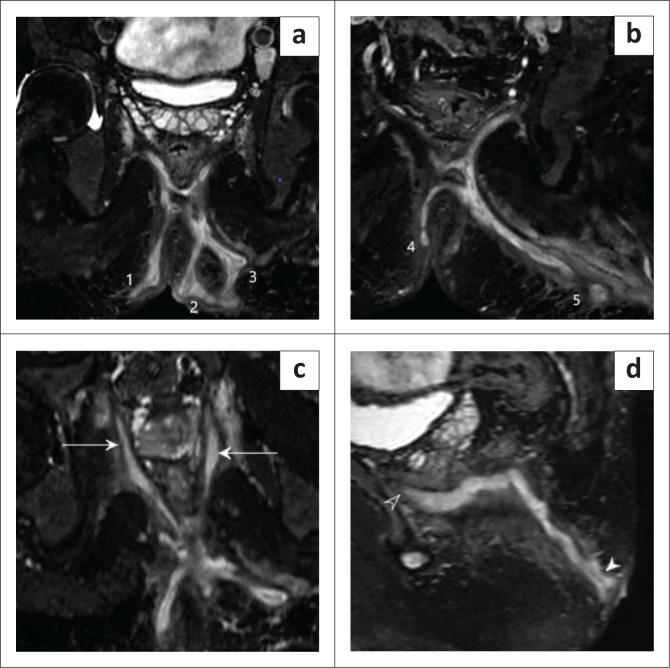
Coronal and sagittal T2 FSE-weighted image post-aqueous gel instillation demonstrating: (a, b) multiple hyperintense intercommunicating fistulous tracts in the ischioanal and ischiorectal fossae with multiple external openings (1, 2, 3, 4 and 5); (c) Axial T2 FSE-weighted image demonstrating a few ramifications noted extending along the levator ani muscle, abutting the prostate and obturator internus muscles on both sides (solid arrows); (d) Sagittal T2 FSE-weighted image demonstrating supralevator extension (hollow arrowhead) – St. James’s Classification Grade 5 fistulous tract.

This study demonstrated that MRF with aqueous gel instillation detected supralevator extension in all three grade 5 cases (Figure 6).

Table 1 documents the surgical correlates with the MRI findings in this study.

**TABLE 1:** Surgical correlates with the MRI findings in this study (*N* = 40).

Findings	MRF with aqueous gel instillation	Intraoperative finding
**Internal opening**		
No	3	0
Yes	37	40
**Secondary tracts**		
No	24	22
Yes	16	18
**Abscess**		
No	20	19
Yes	20	21
**Supralevator extension**		
No	37	37
Yes	3	3

MRF, Magnetic resonance fistulography.

## Discussion

A total of 40 patients with fistula-in-ano were studied, both with and without aqueous gel instillation, ranging in age from 15 years to 60 years, with a mean age of 34.65 years. Among them, 28 were male and 12 were female patients. Anal fistulas predominantly affected young adults, with a higher incidence observed in male patients. The study by Mahakalkar et al.^19^ also demonstrated a male predominance, with 80% of cases occurring in men, resulting in a male-to-female ratio of 4:1. The most affected age group in their study was 41 years – 50 years.

Using the St. James’s classification system, cases were categorised based on their complexity (Figure 1). Grade 1 was the most frequently observed type, identified in 12 patients. This distribution highlights that lower-grade fistulas (Grade 1 and Grade 2) were more frequent, comprising 47.5% of cases, whereas higher-grade fistulas (Grade 4 and Grade 5), which are more complex and challenging to manage, accounted for 32.5% of cases. A study by H. A. I. P. Badder,^20^ analysed MRI findings in 50 patients with clinically confirmed anal fistulas. They documented 30% of cases as Grade 1, 38% Grade 2, 10% Grade 3, 10% Grade 4 and 12% Grade 5, also noting lower-grade fistulas as more common, but that Grade 2 fistulas were more frequent than Grade 1.

Internal openings were identified in 37 cases on MRI with aqueous gel instillation, with the most common locations being between the 5 o’clock and 7 o’clock positions. During surgery, however, internal openings were found in all 40 patients (Table 1). The three cases where internal openings were not detected on MRF were later identified at the 5 o’clock, 6 o’clock and 8 o’clock positions during surgery. In this study, the most frequent location of the internal opening was at the 7 o’clock position (10 cases), followed by the 6 o’clock position (7 patients). Identifying the level of the internal opening on MRI is crucial, as it determines the extent of sphincter division required during fistulotomy. Stoker et al.^21^ reported that T2-weighted and STIR images effectively visualised the internal opening, with findings consistent with surgical observations.

MRI correctly identified 16 of the 18 patients with secondary tracts, yielding a high sensitivity. However, it also accurately identified 22 of the 24 patients without secondary tracts, contributing to a high specificity. The overall accuracy of MRI in detecting secondary tracts was 95%, reflecting its reliable diagnostic capability, though there were slight discrepancies in the identification in some cases.

Abscesses were identified in 20 cases by MR fistulography with gel, while 21 cases were confirmed intraoperatively. In one case, a small abscess was missed by MR fistulography. Additionally, in one case, an internal haemorrhoid was overinterpreted as an abscess by MR fistulography, but it was confirmed to be a haemorrhoid during surgery. These findings highlight the limitations of MR fistulography in accurately distinguishing between abscesses and other conditions, such as lymph nodes or haemorrhoids.

Supralevator extension was identified in three cases by MR fistulography with and without gel, and the same number was confirmed intraoperatively. In spite of the limited sample size of three cases, the study demonstrates good precision. However, the small sample size, short 6-month study duration and low prevalence of supralevator extension limit the ability to draw broader conclusions.

These findings reinforce the superiority of MRI with aqueous gel instillation in detecting internal openings, secondary tracts, abscesses and supralevator extension, thereby enhancing preoperative planning and reducing misdiagnosis. This study demonstrated that the use of aqueous jelly as a contrast medium significantly improved the visualisation of perianal fistulous tracts on MRI. The technique showed high sensitivity and specificity, making it a cost-effective alternative to conventional contrast agents. It provided a clear delineation of internal openings, secondary extensions and lateral ramifications, potentially enhancing surgical planning and reducing recurrence rates. Additionally, aqueous jelly was well tolerated by patients, with no reported post-procedural complications.

Specifically, internal openings were identified with a sensitivity of 92.5%, specificity of 100% and accuracy of 97.5%, closely aligning with the findings of Kumar et al.^22^, who reported a sensitivity of 96.67% and an accuracy of 85% for Grade 1 and 2 intersphincteric fistulas using jelly-enhanced MRI. The detection of secondary tracts in the current study yielded slightly lower but still robust values (sensitivity 88.89%, specificity 91.67% and accuracy 95%), consistent with the results of Aggarwal et al.^4^, who demonstrated 100% sensitivity and specificity with aqueous gel. In terms of abscess detection, the determined sensitivity of 95.24%, specificity of 95% and accuracy of 97.5% paralleled the findings of Al-Khawari et al.^23^, who observed that post-jelly MRI enhanced tract visualisation and provided efficacy comparable to contrast-enhanced MRI, which only added value in 23.3% of cases. Most notably, the current study reported perfect diagnostic performance in detecting supralevator extensions in 3 cases, emphasising the technique’s strength in identifying complex fistula components. The findings not only align with existing literature but also support the routine use of aqueous gel instillation as a standard adjunct in the comprehensive, non-invasive assessment and surgical planning of perianal fistulas.

MRI with aqueous gel instillation delivers excellent visualisation of perianal fistulas and achieves diagnostic accuracy comparable to, or greater than, that of intravenous gadolinium-enhanced MRI, without the need for contrast administration.^24^ This makes it especially advantageous for patients with renal impairment or contrast sensitivity. As a non-invasive, cost-effective alternative, it is particularly useful for individuals who cannot tolerate contrast agents. In comparison, normal saline instillation, while safe, offers poorer contrast and is less effective in visualising complex fistulas. Both gadolinium and saline, because of their watery consistency, only cause temporary distension of the fistulous tracts. Consequently, smaller tracts, the location of internal openings and secondary ramifications may not be sufficiently visualised or reliably identified with these methods. Compared to saline instillation, aqueous gel offers superior tract distension and contrast, thereby enhancing visualisation of small tracts and internal openings.^25^ Additionally, aqueous jelly is cost-effective and accessible, particularly in resource-limited settings. It eliminates the need for expensive contrast agents, reducing financial burdens on healthcare systems. The method is also minimally invasive, well tolerated and easy to perform, making it a practical alternative for fistula imaging.

Comparisons with other imaging modalities also support the superiority of MRI. Sayed et al.^11^ reported that endoanal ultrasound was more effective for locating internal openings, but MRI had better accuracy for detecting fibrotic tracts and secondary extensions. Additionally, Soker’s^26^ study showed that MRI (92.7%) outperformed CT (73.1%) in fistula classification and detection. Garg et al.^27^, in a large study of 229 patients, highlighted MRI’s exceptional sensitivity (98.8%) and specificity (99.7%) in identifying tracts and classifying complex fistulas, further reinforcing the current study’s conclusions.

However, certain limitations should be considered. The relatively small sample size may limit the generalisability of the study findings, necessitating larger, multicentric studies for validation. Additionally, the lack of long-term follow-up data on recurrence rates prevents a comprehensive assessment of the technique’s long-term impact on patient outcomes. Another limitation is the potential for interobserver variability in MRI interpretation, which may influence diagnostic accuracy across different radiologists and institutions.

This study highlights the potential for aqueous jelly-enhanced MR fistulography to become a widely used alternative to traditional contrast methods, particularly in settings with limited access to intravenous contrast agents. Future research should focus on conducting larger, multicentre studies to confirm these findings and establish standardised protocols for its use. Additionally, long-term follow-up studies are needed to evaluate its impact on recurrence rates and post-operative outcomes. Further research on interobserver variability would also help ensure consistency in MRI interpretation across different healthcare settings. If integrated into routine clinical practice, aqueous jelly-enhanced MR fistulography could improve cost efficiency, expand access to high-quality fistula imaging and enhance surgical decision-making, ultimately benefiting both patients and healthcare systems.

## Conclusion

This study demonstrates that MRI with aqueous gel instillation significantly enhances the visualisation of perianal fistulous tracts compared to MRI without gel. The technique improves the detection of internal openings, secondary tracts and abscesses, with high sensitivity, specificity and accuracy. These findings reinforce the superiority of aqueous gel-enhanced MR fistulography as a cost-effective, well-tolerated, and practical alternative to traditional contrast methods.

By improving diagnostic precision, this approach enhances preoperative planning and may contribute to reduced recurrence rates. The findings highlight the potential for aqueous jelly-enhanced MR fistulography to be a promising and cost-effective imaging modality that improves diagnostic accuracy while reducing healthcare costs. Its accessibility makes it particularly valuable in resource-limited settings. However, further large-scale, multicentric studies are needed to validate these results, assess long-term benefits and establish standardised clinical protocols. If widely implemented, this technique could significantly improve patient care and surgical decision-making.
